# Relapse of diabetic ketoacidosis secondary to insulin pump malfunction diagnosed by capillary blood 3-hydroxybutyrate: a case report

**DOI:** 10.4076/1757-1626-2-8012

**Published:** 2009-08-05

**Authors:** John Scott Baird

**Affiliations:** Department of Pediatrics, Columbia UniversityNew York, NY 10032-3784USA

## Abstract

A 14 year old female with type 1 diabetes mellitus and a subcutaneous insulin pump was treated for diabetic ketoacidosis presumed secondary to dietary indiscretion, and then restarted her subcutaneous insulin pump after exchanging the tubing. An hour later, nursing review determined that she was using outdated insulin in the pump, and it was exchanged. However, 5 hours later relapse was suggested by a rise in capillary blood 3-hydroxybutyrate, in spite of a normal serum anion gap and a minimal increase in serum bicarbonate. Insulin pump failure was suspected, and the patient was treated for relapse of diabetic ketoacidosis. Following resolution, her insulin pump was replaced without further complications. Capillary blood levels of 3-hydroxybutyrate may be sensitive, early indicators of relapse of diabetic ketoacidosis, and are easily obtained.

## Introduction

A capillary blood assay for 3-hydroxybutyrate (BHB) has been used at home [1], in hospital emergency departments [2,3] and during hospitalization [4-7] to diagnose and help manage patients with diabetic ketoacidosis (DKA). Capillary blood BHB tests have been used to document the persistence of ketones even while the urine nitroprusside test for ketones showed their clearance during therapy for DKA [8], and one might predict that these capillary blood BHB tests would have utility in detecting relapse following therapy for DKA. Nevertheless, capillary blood BHB testing is not yet routinely available in all children’s hospitals.

An adolescent with a subcutaneous insulin pump and DKA is reported with an early relapse due to insulin pump malfunction, diagnosed by an increase in capillary blood BHB prior to any increase in the anion gap or decline in serum bicarbonate. This case provides further support for the need to monitor capillary blood BHB during therapy for pediatric DKA.

## Case presentation

**Figure 1.**
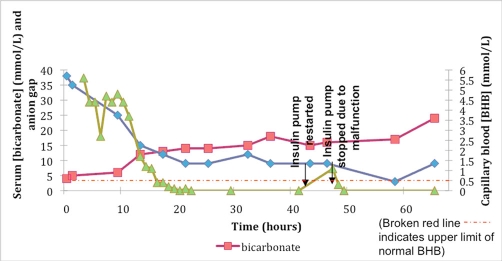
Serum [bicarbonate] and anion gap (AG) with capillary blood [BHB] in patient with DKA and subcutaneous insulin pump.

A 14 year old Caucasian female (United States citizen) with type 1 diabetes mellitus and therapy with a subcutaneous insulin pump for a year, was admitted with severe [9] DKA (arterial blood gas pH 6.96), with serum glucose: 816 mg/dL, PaCO_2_: 17 mmHg, serum anion gap: 38, and 3+ ketones on serum nitroprusside test. She noted dietary indiscretion. Additional laboratory data on admission included serum sodium 120 mmol/L, potassium 5.1 mmol/L, chloride 78 mmol/L, bicarbonate <5 mmol/L, blood urea nitrogen 40 mg/dL, creatinine 1.2 mg/dL, and calcium 10.2 mg/dL. After written informed consent and with IRB approval (University Hospital, University of Medicine and Dentistry of New Jersey in Newark, New Jersey), hourly testing of capillary blood BHB (using the Precision Xtra System for BHB; Abbott Laboratories, MediSense Products Inc; Bedford, MA) was performed concurrently with glucose (fingerstick) testing (Figure 1). Her insulin pump was discontinued, and she received a continuous infusion of intravenous insulin (0.1 to 0.14 units/kg/hr) and rehydration. The serum anion gap and capillary blood BHB fell to normal levels (<12 and <0.5 mmol/L, respectively) at 14 hours. Due to persistent metabolic acidosis, the insulin infusion was continued for a total of 42 hours, when serum [bicarbonate] was 18 mmol/L. She felt much better and resumed her regular diet and therapy with her subcutaneous insulin pump (after changing all pump tubing).

Serum bicarbonate fell to 15 mmol/L while serum chloride increased to 116 mmol/L (serum anion gap: 9) less than 1 hour later. Subsequently, she had a meal as well as a snack. Within an hour of restarting the insulin pump, nursing staff determined that the patient had been using outdated insulin, and this was rectified. She was given several extra doses of subcutaneous (regular) insulin over the next 4 hours. Five hours after discontinuation of the intravenous insulin infusion, serum bicarbonate rose slightly to 16 mmol/L and the anion gap was 9, though hyperglycemia recurred (serum glucose: 501 mg/dL).

Simultaneously, capillary blood BHB rose to 1.1 mmol/L. The insulin pump was again stopped and the patient received 20 more hours of intravenous insulin infusion and hydration; her serum bicarbonate was then 24 mmol/L with euglycemia. The pump was then replaced due to a malfunction, and the patient was discharged home without further complications.

## Discussion

An episode of DKA in a patient with a subcutaneous insulin pump mandates a detailed review to discover the cause: in this patient, a dietary indiscretion was initially suspected, though outdated insulin was subsequently noted and exchanged. However, mechanical pump failure complicated this case, and was responsible for relapse shortly following resumption of a normal diet.

At the time relapse was diagnosed, the serum bicarbonate had increased slightly with concurrent hyperchloremic metabolic acidosis and postprandial hyperglycemia. Hyperchloremic metabolic acidosis may be associated with DKA, either on presentation or as a result of therapy [10], as in this patient. Nevertheless, the capillary blood BHB was also tested and confirmed relapse of DKA [11] secondary to subcutaneous insulin pump malfunction. Progressive ketosis can develop soon after interruption of a continuous insulin infusion in diabetics [12]. It is likely that recognition of DKA relapse in this patient would have been delayed in the absence of capillary blood BHB testing.

Interest and experience with subcutaneous insulin pump therapy is increasing [13], and practical experience with the management of these patients while hospitalized may be helpful. Multiple triggers for DKA may be present in diabetic children treated with a subcutaneous insulin pump, as in this patient. Capillary blood BHB is helpful in detecting relapse: a fingerstick BHB level is easily obtained at the bedside in conjunction with a glucose level and does not entail significant delays in processing. Capillary blood BHB levels have been recommended as routine for managing sick children with diabetes [1], and this technology “is a useful adjunct to laboratory-based determinations” according to a consensus statement from the American Diabetes Association [9]. It may be particularly useful for monitoring sick children with diabetes and a subcutaneous insulin pump [14].

## Abbreviations

BHB: 3-hydroxybutyrate
DKA: diabetic ketoacidosis
IRB: institutional review board
